# Microbiota and Radiotherapy: Unlocking the Potential for Improved Gastrointestinal Cancer Treatment

**DOI:** 10.3390/biomedicines13020526

**Published:** 2025-02-19

**Authors:** Damir Vučinić, Arnela Redžović, Goran Hauser, Ivana Mikolašević

**Affiliations:** 1 Tumor Clinic, Clinical Hospital Centre Rijeka, Krešimirova 42, 51000 Rijeka, Croatia; arnelaredzovic2266@gmail.com (A.R.); ivana.mikolasevic@gmail.com (I.M.); 2 School of Medicine, University of Rijeka, 51000 Rijeka, Croatia; goran.hauser@medri.uniri.hr; 3 Department of Gastroenterology, Clinical Hospital Center Rijeka, Krešimirova 42, 51000 Rijeka, Croatia

**Keywords:** microbiota, radiotherapy, gastrointestinal cancer, dysbiosis, short-chain fatty acids

## Abstract

Radiotherapy (RT) is one of the major cornerstones in managing gastrointestinal (GI) cancers. However, several side effects, such as intestinal inflammation, mucosal injury, and dysbiosis, often compromise this. The gut microbiota increasingly attracts much interest as an essential modulator of RT effects influencing immune responses and tissue repair. Through short-chain fatty acids such as butyrate, representatives of certain bacterial species play a crucial role under normal conditions, keeping the mucosal integrity intact and reducing oxidative stress-mediated damage. Dysbiosis, a state where diminished microbial diversity and increased pathogenic species in the microbiota are seen, amplifies RT-induced toxicity in patients. Clinical investigations highlight that microbiota-targeted interventions, including probiotics, prebiotics, and fecal microbiota transplantation, hold the means to augment RT efficacy and lessen toxicity. Increased microflora diversity and specific microbial profiles have yielded serious patient improvements. Advanced RT methods use stereotactic body radiotherapy combined with microbiota modulation as a promising technique to shield healthy tissue and maximize immune-mediated antitumor effects. Additionally, there is an implication in tumor behavior regulated by the intratumoral microbiota regarding the response to radiotherapy. Notably, the modulation of gut and tumor microbiota provides an avenue to optimize RT benefits in GI cancers, underscoring the importance of personalized therapy.

## 1. Introduction

Radiotherapy (RT) is one of the fundamental methods for treating solid tumors. By precisely targeting ionizing radiation to the tumor area, it damages cancer-cell DNA and promotes tumor cell death or loss of proliferative capacity [1]. Despite technological advancements in optimizing dose delivery and targeting, patients frequently experience adverse effects such as intestinal inflammation, nausea, and diarrhea, which can limit the application of high-efficacy radiation doses [2]. Over the past ten years, more and more studies have examined how the microbiota affects how the body reacts to different cancer treatments, such as radiation therapy [3,4]. The gut microbiota, a complex population of microorganisms, can affect RT results and safety because it affects various physiological functions, from immune response regulation to mucosal integrity maintenance [5]. However, interindividual variability in microbiota composition and function remains a significant challenge in standardizing microbiota-based interventions. Understanding how to modulate these microbial communities to maximize therapeutic benefits while minimizing potential risks is a key consideration [4]. Short-chain fatty acids (SCFAs), including butyrate, are among the metabolites produced by microorganisms that influence inflammation and aid in tissue healing [6]. In a study by Zheng et al., adding a butyrate-producing probiotic formulation during RT improved mucosal integrity. It resulted in a notable decrease in gastrointestinal side effects, reflecting the essential role of SCFAs in maintaining gut homeostasis under oxidative stress [7]. By stimulating T-lymphocytes and dendritic cells, specific gut microbial compositions may also improve the anticancer effects of RT [3,4,8]. On the other hand, dysbiosis can lead to acute and chronic damage and make healthy tissues more vulnerable to radiation [9]. Research indicates that patients with a more diverse gut microbial community are at a higher risk for tissue damage and gastrointestinal adverse events compared to those with a more stable microbiota [10]. This suggests that while microbiota diversity is generally beneficial, an imbalance under RT-induced stress may contribute to toxicity, necessitating careful monitoring and potential microbiota-targeting interventions. These data accentuate the need to monitor and correct dysbiosis for optimal radiotherapy results. Modifying the gradual gut microbiota through probiotics, prebiotics, synbiotics, or fecal microbiota transplantation may boost the efficacy of radiotherapy and reduce adverse side effects [4,11]. A recent meta-analysis by Danis et al. showed a noteworthy decrease in gastrointestinal toxicities and an improvement in the therapeutic outcome for patients receiving both probiotics and prebiotics together with radiotherapy [12]. The scientists highlighted the value of a customized strategy when choosing suitable strains and figuring out the ideal dosage and treatment period by contrasting different probiotic formulations and research procedures. More thorough clinical research is necessary to validate such therapies’ best practices, dosage, and duration despite promising first results [13].

This study aims to systematically evaluate the impact of gut microbiota modulation on RT outcomes, mainly focusing on its role in enhancing therapeutic efficacy. By integrating clinical and preclinical data, we seek to identify microbiota-based strategies that could serve as adjunctive therapies in personalized radiotherapy protocols.

### 1.1. Modulation of the Microbiota in Oncological Therapy

Gut microbiota modulation is increasingly recognized as a key factor in cancer therapy because intestinal microorganisms affect the host immune system and tumor therapy outcomes. This community of bacteria, fungi, and viruses interacts with the immune system through complex mechanisms of inflammation regulation, metabolism, and the maintenance of the intestinal barrier [3,5]. Many studies indicate that a balanced or “healthy” microbiota can enhance immunotherapy effectiveness, whereas dysbiosis can exacerbate inflammation and promote tumor progression [8,9]. Among the key mechanisms by which the microbiota participates in antitumor responses is the production of SCFAs. Specific bacterial genera, such as *Bifidobacterium* and *Faecalibacterium*, ferment dietary carbohydrates to produce SCFAs, most notably butyrate [5,6]. Butyrate has anti-inflammatory and immunomodulatory properties, supporting regulatory T-lymphocyte (Treg) differentiation and balancing Th1/Th2 immune responses. The Sanchez and Liu study demonstrated that specific gut microbiota metabolites, particularly butyrate, are pivotal in enhancing Treg differentiation and promoting an immunosuppressive tumor microenvironment [11,13]. While some studies suggest that patients with a higher abundance of *Faecalibacterium* and *Bifidobacterium* tend to experience improved therapeutic outcomes, it is important to acknowledge that responses to microbiota modulation are influenced by multiple factors, including the tumor microenvironment, host immune status, and individual genetic predisposition. Not all patients exhibit the same degree of benefit; in some cases, specific microbial compositions may even interfere with treatment efficacy [9,10]. The microbiome affects several molecular pathways that support tumor immune regulation and SCFAs. Certain bacterial species can convey tumor antigens to T-lymphocytes more effectively by stimulating dendritic cell activation or increasing the production of components involved in antigen presentation [14]. Microbial metabolites can also modulate macrophage and natural killer cell activity via Toll-like receptors, thus promoting more robust antitumor responses. Clinical findings show that the efficacy of immune checkpoint inhibitors (e.g., PD-1 and CTLA-4) can be improved by modulating the microbiota [3,12]. For instance, melanoma patients with a highly diverse gut microbiota and elevated levels of beneficial bacteria exhibited better responses to anti-PD-1 therapy and longer progression-free intervals [8]. The therapeutic manipulation of the microbiota carries potential risks and limitations. Interventions may have variable effects depending on individual microbiota composition, making it difficult to standardize treatment approaches. Excessive or unintended alterations in the microbiota could disrupt immune homeostasis, leading to unintended consequences such as increased inflammation or reduced efficacy of immune therapies. Therefore, careful patient selection and monitoring are necessary to optimize benefits while minimizing potential risks [12,13]. Future research should focus on identifying biomarkers that predict patient responses to microbiota-targeted therapies, enabling the development of personalized treatment strategies. Personalized treatment approaches considering the tumor genome, microbiota composition, and the patient’s overall condition could become integral to standard oncology practice [5,14].

### 1.2. Interaction Between the Immune System and the Microbiota During Radiotherapy

**Figure 1:**
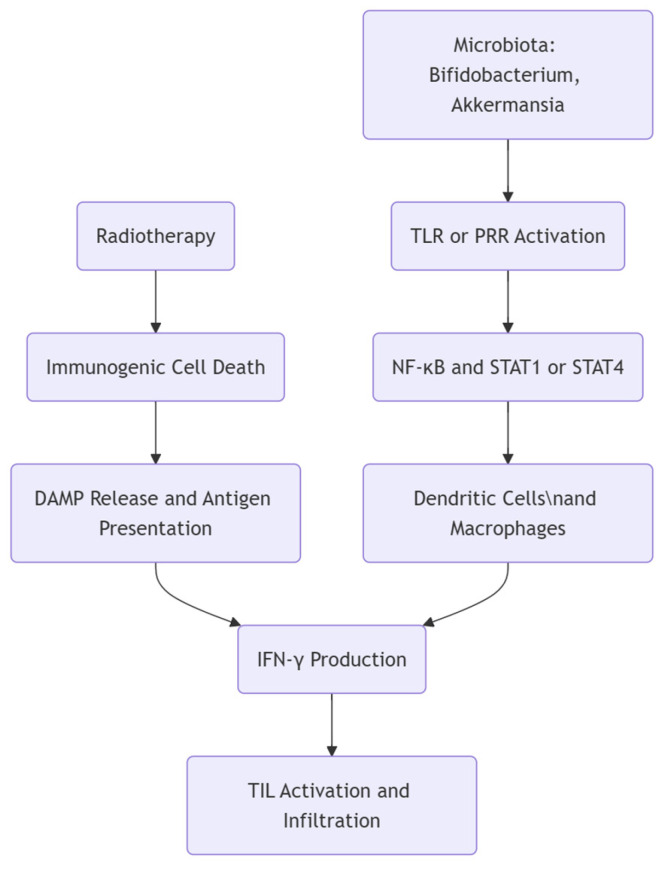
Schematic representation of the interplay between the microbiota, RT, and TILs in tumor immunity. The microbiota, including *Bifidobacterium* and *Akkermansia*, activates pattern recognition receptors (PRRs) such as Toll-like receptors (TLRs), leading to downstream activation of nuclear factor kappa B (NF-κB) and signal transducer and activator of transcription (STAT1/STAT4) pathways. This stimulates dendritic cells (DCs) and macrophages, which enhance IFN-γ production, a key cytokine for TIL activation and infiltration. RT induces immunogenic cell death, releasing danger-associated molecular patterns (DAMPs) that promote antigen presentation, further contributing to IFN-γ production and TIL recruitment into the tumor microenvironment.

The interplay between immunity and the microbiota during RT is pivotal in determining clinical outcomes and potential side effects. From a radiobiological perspective, RT damages DNA in tumor cells and influences immune cells and the microbiota [1,4]. Damage to the intestinal epithelium and local tissue changes can disrupt the balance between beneficial and pathogenic microbes, promoting inflammation that may further shape antitumor immune responses [3,9]. Tumor-infiltrating lymphocytes (TILs) play a crucial role in mediating immune responses within the tumor microenvironment (TME), and recent evidence suggests that microbiota modulation can influence TIL recruitment and activity [5]. Specific bacterial species, such as *Bifidobacterium* and *Faecalibacterium*, have been associated with enhanced TIL activation through increased antigen presentation and cytokine signaling. One of the key cytokines influenced by the microbiota in relation to TIL activity is interferon-gamma (IFN-γ). IFN-γ is primarily produced by activated CD8^+^ cytotoxic T-lymphocytes and CD4^+^ Th1 cells, and its production can be enhanced by microbiota-driven stimulation of dendritic cells (DCs) and macrophages [8,14]. Certain gut bacteria, such as *Bifidobacterium* and *Akkermansia muciniphila*, can promote IFN-γ production by activating pattern recognition receptors (PRRs), including Toll-like receptors (TLRs), leading to downstream activation of nuclear factor kappa B (NF-κB) and STAT1/STAT4 pathways. RT can further amplify IFN-γ responses by inducing immunogenic cell death (ICD), which releases tumor-associated antigens and danger-associated molecular patterns (DAMPs), leading to dendritic cell activation and enhanced antigen presentation to T-cells [1,6,9] (Figure 1). However, these effects can vary depending on tumor type, immune status, and the overall composition of the microbiota, highlighting the need for personalized approaches in microbiota-based therapies.

**Figure 2:**
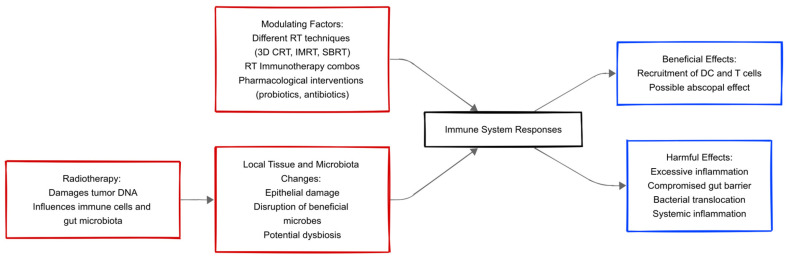
The figure illustrates the interplay among radiotherapy, the local tissue environment (including the gut microbiota), immune system responses, and factors that can modulate treatment outcomes.

Healthy lymph nodes, sufficient vasculature, and TILs significantly impact how well immunotherapy and radiation therapy work together. Because TILs can identify and combat cancer cells, they are essential to the immune response against tumors. While functional lymph nodes enable efficient immune surveillance and response, a well-developed vascular network guarantees the appropriate delivery of immune cells and therapeutic substances to the tumor site. Recent studies demonstrate that specific gut microbiota profiles are associated with improved responses to radiotherapy and immunotherapy combinations, mainly through enhanced TIL activation and cytokine signaling within TME [15]. On the one hand, localized inflammation may recruit dendritic cells and T-lymphocytes, leading to the so-called “abscopal effect”, where antitumor activity extends beyond the irradiated region [16]. The authors demonstrated that modulating the gut microbiota using oral vancomycin significantly enhanced the systemic anti-tumor immune response induced by radiotherapy. The study showed increased activation of dendritic cells (CD11c^+^MHCII^+^) and cytotoxic T-lymphocytes (CD8^+^), as well as reduced populations of regulatory T-cells (Tregs), resulting in improved tumor regression at both irradiated and distant sites. When combined with immunotherapy, the authors highlighted the potential for gut microbiota manipulation to enhance abscopal effects [17,18]. Uribe-Herranz et al. found that fecal microbiota transplantation (FMT) in combination with anti-PD-1 immunotherapy improved radiotherapy outcomes in preclinical colorectal cancer models. The levels of cytokines involved in inflammatory processes, IFN-γ and TNF-α, were elevated in preclinical models. Specifically, the mice exposed to both FMT acceptability for donation and immunotherapy with anti-PD1 demonstrated a 2.5-fold increase in IFN-γ levels and a 1.8-fold increase in TNF-α within the tumor microenvironment when compared to controls, reflecting a higher systemic anti-tumor immunity [19]. More specifically, T-cell responses and tumor immune control, perhaps mediated through tumor microenvironment regulation, are associated with specific bacterial species of the gut microbiota, such as *Bifidobacterium* spp. and *Faecalibacterium prausnitzii,* and they correlate well with better therapeutic success in tumor eradication [3,6]. Despite these promising findings, microbiota modulation is not without risks. Alterations in the microbiota composition could lead to unintended immune dysregulation, potentially exacerbating inflammation or even promoting immune tolerance in specific tumor contexts [4]. Different RT methods, such as conformal RT, intensity-modulated RT (IMRT), and stereotactic ablative radiotherapy (SABR/SBRT), maximize tumor control but minimize doses to healthy tissues [2]. Even the most accurate methods can potentially irradiate parts of the colon, changing the microbiota. The total dosage, fractionation, and amount of irradiated tissue affect how much these alterations occur. For instance, compared to traditional RT schedules, SABR may reduce collateral damage to neighboring tissues by delivering high doses in fewer fractions across a smaller volume [20]. The importance of intratumoral microbiota—the bacteria that live inside tumor tissues—in affecting the course of cancer and its response to treatments, such as SBRT, has recently come into focus. The intratumoral microbiota of several cancer types, including lung cancer, which is frequently treated with SBRT, was examined by Lombardo et al. The researchers found that specific microbial signatures were present depending on the type of tumor, suggesting that the intratumoral microbiota could influence tumor behavior and response to treatments [17]. Research indicates that different radiation doses can affect immune function and bacterial dynamics. Sublethal doses may sometimes enhance immunostimulatory activity by increasing neoantigen expression, whereas higher doses can accelerate tissue destruction and dysbiosis, potentially leading to secondary infections or immune exhaustion [21]. Finally, the balance between beneficial and detrimental RT effects on the immune system and the microbiota can be fine-tuned by pharmacological interventions (e.g., probiotics, prebiotics, targeted antibiotics). (Figure 2) However, caution is warranted when implementing such strategies, as an overly aggressive approach to microbiota modulation could disrupt host-microbe homeostasis, leading to unforeseen complications.

### 1.3. Effects of Radiotherapy on the Gut Microbiota

Though it also impacts other body systems, such as the gut microbiome, RT mainly works by directly harming the DNA of tumor cells [2]. Radiation does not selectively target malignant cells alone; it can also damage the intestinal mucosa, altering the environment in which bacteria reside. As a result, potentially harmful species multiply while helpful bacterial strains frequently decline [5]. This imbalance can result from an enhanced inflammatory response, disruption of the gut barrier, and a higher risk of gastrointestinal adverse effects such as discomfort, diarrhea, and mucositis [10]. Because of its rapid cellular turnover, the gut lining is especially vulnerable to radiation-induced damage. RT compromises the integrity of the epithelial barrier, disrupts mucosal immunity, and creates an unfavorable environment for beneficial bacteria like *Lactobacillus* and *Bifidobacterium*, which rely on a healthy epithelial layer and nutrient availability. At the same time, opportunistic bacteria like *Clostridium* and *Enterococcus* have a chance to multiply due to the changed environment, which is marked by inflammation, impaired immunological signaling, and necrotic tissue [6,7,22]. Animal models have provided insights into these mechanisms. For instance, RT-induced dysbiosis in mice has been linked to damage in organs beyond the gut, possibly through altered gut–lung cross-talk [10,20]. Other studies found that introducing certain compounds (e.g., hyaluronic acid) can have radioprotective effects by modulating gut microorganisms [22]. SCFAs maintain the integrity of the epithelium and immune regulatory mechanisms at the cellular level, which is especially important in the radiation-induced oxidative stress environment [6].

The authors of two recent studies discussed the dual role of oxidative stress, including its ability to promote tumor suppression and recurrence, depending on the context and level of stress [20,21]. RT primarily exerts its effects by generating oxidative stress in targeted tissues, ionizing radiation interacts with water molecules in cells to produce free radicals, such as hydroxyl radicals (•OH) and superoxide anions (O_2_^−^), which cause extensive DNA damage in cancer cells, leading to cell death [9]. A study by Yi et al. provides new insights into the effects of oxidative stress on the gut microbiota. In a mouse model, the researchers studied how reactive oxygen species (ROS) produced during radiotherapy affected the gut microbial composition. They discovered a notable decline in microbial diversity, especially in bacteria that produce SCFAs, like *Roseburia* and *Faecalibacterium* [23]. Riehl et al. investigated strategies to mitigate RT-induced oxidative stress and its impact on the microbiota. Their study revealed that microbiota-targeted interventions, such as probiotics promoting butyrate production, can partially restore balance by enhancing epithelial repair and reducing inflammation [22]. The direct effects of RT on bacterial DNA in the gut microbiota have been the subject of numerous investigations, emphasizing how radiation-induced damage affects bacterial survival, replication, and metabolic activity. According to studies, radiation damages bacterial DNA by causing severe double-strand breaks (DSBs), which reduces the survival of beneficial microbes like *Lactobacillus* and *Bifidobacterium* and hinders their capacity to carry out vital tasks, including immune regulation and SCFA production [24]. Concurrently, radiation-induced DNA damage can drive mutagenesis in certain bacterial species. For example, *Clostridium difficile* has been shown to develop adaptive mutations post-RT exposure, increasing its pathogenicity and oxidative stress resistance and exacerbating dysbiosis [25]. A vast difference in the sensitivity of bacterial species to radiation can be found, not only due to differences in the DNA repair mechanisms but also due to structural and subsequent metabolic adaptations. Gram-negative bacteria, such as *Escherichia coli*, possess greater cell wall resilience due to the outer membrane acting as a barrier against oxidative stress and radiation-induced damage. The outer membrane contains lipopolysaccharides (LPSs), which further decrease the direct exposure of the bacterial cell wall to ROS, decreasing the rate of lipid peroxidation and, therefore, membrane disruption [21,25]. Also, Gram-negative bacteria initiate the SOS response, a well-characterized DNA damage repair system that augments radiation repair of double-strand breaks and survival under genotoxic stress. In contrast, Gram-positive bacteria, such as *Lactobacillus* and *Bifidobacterium*, lack this outer membrane and suffer much higher vulnerability to ionizing radiation [4]. The exposed peptidoglycan layer, therefore, means increased susceptibility to oxidative stress, reducing survival and impairing functional capacities post-RT exposure. In addition, the differing local expression of antioxidant enzymes, such as catalase and superoxide dismutase, accounted for the variation in resistance among species. Gram-negative bacteria usually give a better defense against oxidative stress [5]. Besides direct DNA damage, RT can also trigger horizontal gene transfer (HGT) among gut microorganisms, impacting antibiotic resistance. The infractions in DNA by radiation set up situations where bacteria engage in heightened genetic exchange through transformation, transduction, and conjugation. It has been shown that RT-induced dysbiosis and inflammation favor the transfer of antibiotic resistance genes (ARGs) among opportunistic pathogens already known to bear resistance against standard antimicrobial therapies such as *Enterococcus* and *Clostridium difficile*. This is particularly pertinent in RT-induced immunocompromised patients, in whom changes in the gut microbiota may allow secondary infections with multidrug-resistant organisms [19,20]. While the relative extent of radiation-driven transfer in the human microbiota is not yet completely unraveled, preclinical evidence suggests gut inflammation and epithelial barrier disruption impacting at least some of the antibiotic-resistance genes by RAT could facilitate gene exchange among the pathogenic bacterial population. There is a need for further studies to ascertain the implications of alteration in the gut microbiota upon the evolution of antibiotic resistance in a clinical setting [26].

## 2. The Impact of Microbiota Modulation on the Treatment of Gastrointestinal Tract Tumors

### 2.1. Microbiota Modulation in Radiotherapy for Esophageal Cancer

Despite improvements in therapeutic approaches, esophageal cancer still poses a serious threat to world health because of its poor survival rates. There is growing evidence that the gut and tumor microbiome may affect how well cancer treatments, such as RT, work. Van den Ende et al. conducted a longitudinal prospective study to examine the microbiota composition of 172 esophageal cancer patients receiving neoadjuvant chemoradiotherapy. Tumor and duodenal biopsies were taken from a subgroup of patients, and fecal samples were taken at baseline, during treatment, and before surgery. The study revealed significant changes in fecal, tumor, and duodenal microbiota profiles. Patients who achieved a pathological complete response exhibited stable fecal alpha diversity, whereas poor responders experienced a decrease in alpha diversity during treatment. Furthermore, lower pre-surgery alpha diversity was associated with worse progression-free survival. Notably, baseline tumor biopsies from patients with shorter progression-free survival showed higher abundances of *Fusobacterium* [27]. These results highlight the possibility of using microbiota composition to predict treatment outcomes and survival in patients with esophageal cancer. Sasaki et al. evaluated the gut microbiota of 51 esophageal squamous cell carcinoma patients receiving chemoradiotherapy. Fecal samples obtained before therapy were examined to assess the microbiome’s composition. According to the study, patients with advanced clinical stages (III-IVb) had a greater relative abundance of *Fusobacteriaceae*. On the other hand, the relative abundance of *Lactobacillaceae* was higher in individuals who responded either fully or partially to chemoradiotherapy [28]. Mechanistically, *Fusobacterium* species have been implicated in promoting a pro-inflammatory tumor microenvironment by activating NF-κB and inducing the production of pro-inflammatory cytokines such as IL-6 and tumor TNF-α. These inflammatory pathways can contribute to immune evasion and tumor progression, potentially explaining the association between *Fusobacterium* and poor treatment responses [6,23]. Conversely, *Lactobacillaceae* are known to exert immunomodulatory effects by enhancing the production of SCFAs and promoting anti-inflammatory responses via increased Treg activation. This may help mitigate treatment-induced inflammation and improve response rates to chemoradiotherapy. Other factors, such as dietary habits, antibiotic use, and tumor burden, may also significantly influence microbiota composition and treatment outcomes [20]. Moreover, antibiotic exposure before or during treatment can drastically alter microbiota composition, potentially reducing beneficial bacterial populations such as *Lactobacillus* and *Bifidobacterium*, which have been associated with improved treatment responses [5]. Tumor burden and stage may further modulate microbiota profiles, as more advanced disease is often linked to increased inflammation and metabolic changes that favor pathogenic microbial expansion [20,29]. Li et al. demonstrated that higher gut microbiome alpha diversity is significantly associated with favorable responses to chemoradiotherapy in patients with esophageal squamous cell carcinoma, including higher tumor regression rates. Lower microbiome diversity, on the other hand, was associated with worse treatment outcomes, indicating that microbiome diversity may be a prognostic indicator and possible target for therapeutic interventions [30]. In patients with advanced esophageal cancer, authors discovered a strong correlation between the intestinal flora’s structure and the effectiveness of chemoradiotherapy, with greater microbial diversity being related to better treatment outcomes. Better treatment results were positively connected with some bacterial species, including *Faecalibacterium* and *Bacteroides* [31]. Squamous cell carcinoma and adenocarcinoma are two types of esophageal malignancies that differ in biology in this research. These differences could significantly impact microbiome profiles and how they affect treatment (Table 1). Future research should discriminate between these subtypes to better understand the involvement of the microbiome in various histological types of esophageal cancer and create customized treatment plans [32].

### 2.2. Microbiota Modulation in Radiotherapy for Pancreatic–Biliary System Cancer

Both benign and malignant pancreaticobiliary diseases exhibit distinct microbiome fingerprints. Authors like Song et al. related that, while progressing from chronic cholecystitis to biliary tumors, dominant bacteria in the gallbladder would be *Peptostreptococcus stomatis, Fusobacterium mortiferum, Acinetobacter junii*, and *Enterococcus faecium* [33,34]. These bacteria may contribute to carcinogenesis by promoting chronic inflammation, disrupting bile acid metabolism, and facilitating DNA damage through microbial metabolites such as hydrogen sulfide and secondary bile acids. *Fusobacterium mortiferum* for example, has been associated with an inflammatory tumor microenvironment via TLR activation, leading to increased IL-6 and TNF production, which can support tumor progression. Similarly, *Peptostreptococcus stomatis* has been implicated in immune evasion by altering antigen presentation and modulating T-cell responses in biliary cancer [35]. The gut microbiota may play a role in developing and progressing hepatocellular carcinoma (HCC). A gut-microbiota-based signature comprising eight genera—*Faecalibacterium*, *Klebsiella*, *Ruminococcus gnavus group*, *Lactobacillus*, *Dorea*, *Veillonella*, *Burkholderia caballeria paraburkholderia*, and *Citrobacter*—has demonstrated high accuracy in distinguishing patients with cholangiocarcinoma (CCC) or HCC [36]. Biliary dysbiosis and specific bile metabolites, such as isoleucine, have been implicated in the development and progression of CCC [37]. Distinct gut microbiota, such as *Ruminococcaceae*, have been inversely associated with chemotherapy response in CCC patients receiving first-line cisplatin and gemcitabine combination therapies [38]. While this correlation is notable, it remains unclear whether *Ruminococcaceae* directly influences chemotherapy efficacy or if chemotherapy itself alters microbiota composition. One possible explanation is that Ruminococcaceae affect drug metabolism by modulating bile acid transformation or detoxification pathways, which may impact drug availability in the TME. Alternatively, chemotherapy-induced damage to the gut lining could favor the expansion of opportunistic bacteria, altering the balance of microbial populations over the course of treatment. Further studies are needed to establish causality and determine whether microbiota modulation can enhance chemotherapy responses. Impaired anti-HCC immunity is associated with gut microbiota dysbiosis, which inhibits antigen-presenting cells and suppresses effector T-cell function [39]. Modulating the gut microbiome presents a novel strategy for enhancing radiosensitivity in liver cancer patients undergoing radiotherapy [16]. Pancreatic cancer is a leading cause of cancer-related deaths. Radiotherapy has been shown to improve local control as an adjuvant therapy due to its cytostatic activity. The gut microbiota of pancreatic cancer responders can metabolize tryptophan from food into indole-3-acetic acid (3-IAA), which inhibits tumor cell autophagy, reducing the tumor cells’ ability to adapt to cellular stress [40]. While the microbiota and their products can influence the pancreatic microenvironment, comprehensive data are lacking. The field of microbiota research is still in its early stages. The specific molecular mechanisms and interactions between radiotherapy and pancreaticobiliary microenvironments remain unclear, necessitating further investigations and future studies to establish correlations and causality. Future studies should explore whether microbiota-targeted interventions, such as probiotics or dietary modifications, could enhance treatment efficacy in pancreaticobiliary cancers.

### 2.3. Microbiota Modulation in Radiotherapy for Rectal Cancer

**Figure 3:**
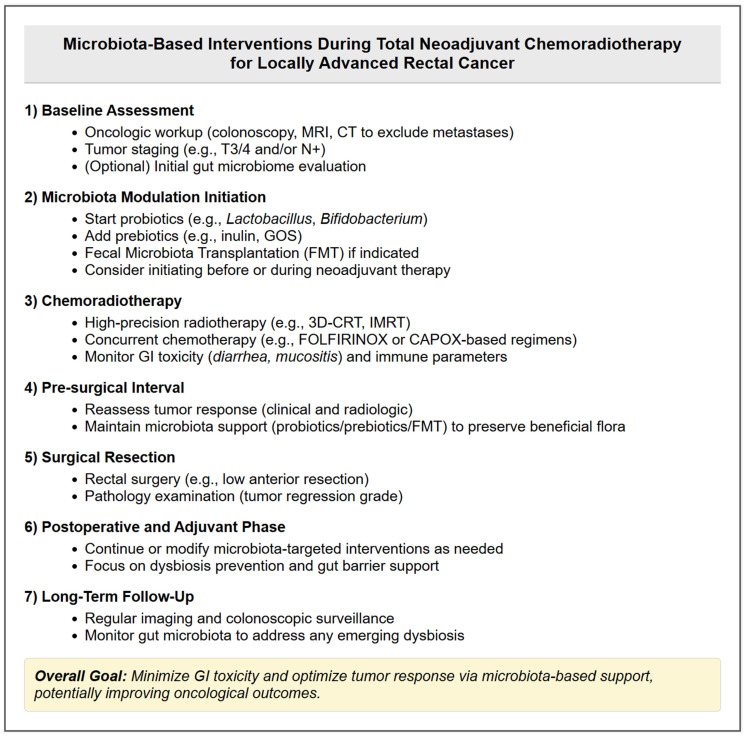
Microbiota-based interventions during total neoadjuvant chemoradiotherapy for locally advanced rectal cancer. This schematic outlines key time points—from baseline assessments, neoadjuvant chemoradiotherapy, and surgery, to the postoperative and long-term follow-up phases—where gut microbiota modulation (via probiotics, prebiotics, or fecal microbiota transplantation) can be implemented. The goal is to help preserve beneficial bacterial strains, reduce gastrointestinal toxicity, and potentially improve oncological outcomes.

**Figure 4:**
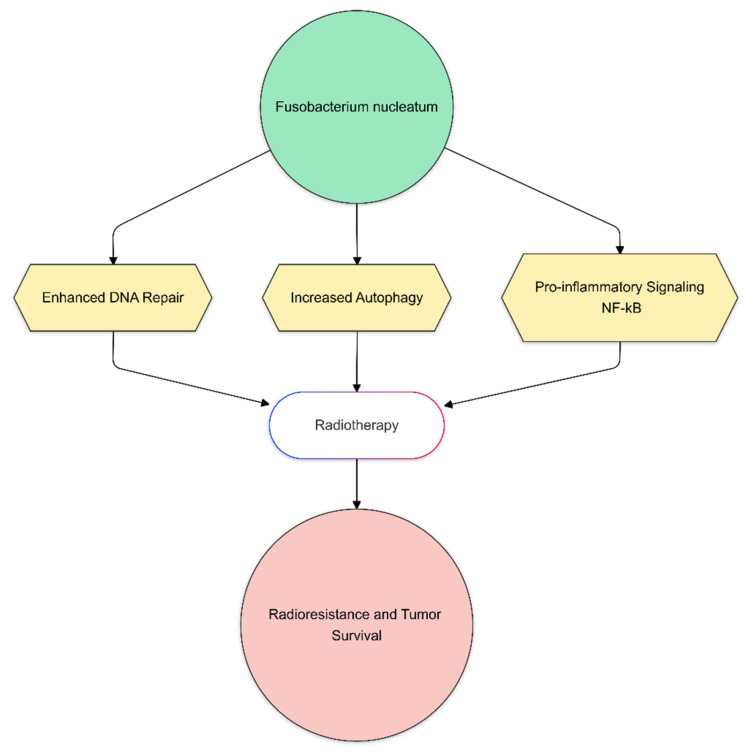
Proposed mechanism by which Fusobacterium nucleatum promotes radioresistance in colorectal cancer. The bacterium enhances DNA repair, increases autophagy, and triggers pro-inflammatory pathways (e.g., NF-kB) in tumor cells, ultimately reducing the effectiveness of radiotherapy.

Colorectal cancer (CRC) is one of the most common cancers, with a high mortality rate worldwide. Biological parameters are closely associated with therapy outcomes [6]. Intestinal flora is of significant interest in anticancer therapy. According to research conducted by the Human Microbiome Project (HMP), founded in 2008, patients with CRC have lower levels of bacterial variety and richness than people in good health. There is strong evidence that the gut microbiota’s makeup influences tumor sensitivity to anticancer treatment and is associated with the development and spread of colorectal cancer [41]. Recent research highlights the gut microbiota’s critical role in modulating radiotherapy responses in rectal cancer patients [42] (Figure 3). Specifically, the better outcomes were associated with specific microbial taxa, such as *Clostridium sensu stricto 1* and *Intestinimonas*, in patients undergoing neoadjuvant chemoradiotherapy (nCRT) with a prediction model giving an AUC of 0.821 [43]. In addition, Sun et al. could also accurately predict nCRT response by identifying key microbial biomarkers, namely Dorea, Anaerostipes, and Streptococcus (AUC 0.9357) [44]. Also, another study found that the responders for nCRT were enriched in *Shuttleworthia*, whereas specific *Clostridiales* taxa were enriched in non-responders [15]. These functional variations link with pathways like fatty acid metabolism that may influence treatment effectiveness. Zhang et al. discovered that neoadjuvant radiation decreased cancer-associated taxa and caused oral infections to move into the gut, which may have consequences for patient health, even if it did not significantly change alpha diversity [45]. Another clinical study has shown that patients receiving pelvic radiotherapy show prominent changes in their gut microbiota, particularly in *Firmicum* and *Fusobacterium*, which have been found to decrease by 10% and increase by 3%, respectively [46,47]. While *Fusobacterium nucleatum* has been associated with chemoresistance in CRC, its role in activating autophagy remains under investigation. Current evidence suggests that *F. nucleatum* may influence autophagy through the activation of TLR4 and the upregulation of microRNAs involved in autophagic pathways, but causality has yet to be firmly established [42,48] (Figure 4). However, no studies have been published to date on the potential effects of gut microbiome composition on radiosensitivity via modulation of autophagy. Moreover, pre-existing inflammation associated with tumor progression can create an environment favoring the expansion of pro-inflammatory bacterial taxa such as *Fusobacterium*, further complicating microbiota-mediated treatment interactions. Pooled results suggest that the inflammatory environment produced by radiotherapy leads to an increased abundance of other pathogenic pro-inflammatory bacteria (e.g., *S. wadsworthensis* and *S. parvirubra*) and a decreased abundance of anti-inflammatory bacteria (e.g., *E. faecalis* and *Prevotella cisticola*) and *Phylum firmicutes* including *Lachnospira pectinoschiza*, *Roseburia intestinalis*, etc. [49,50]. Microbial colony metabolites may activate the STAT3 signaling pathway in immune cells. Specific bacterial metabolites, such as SCFAs, have been shown to modulate immune responses through STAT3 activation. Park SY et al. demonstrated that this pathway plays a role in inflammation-driven radioresistance in CRC patients undergoing RT [47,51]. *Lactobacilli* are the most common genera, and their anticancer properties are due to their different mechanisms, including modification of the intestinal barrier, modulation of the host’s immune responses, induction of apoptosis, and production of metabolites with antiproliferative and anti-inflammatory characteristics [52]. *Lactobacillus* and *Bifidobacterium* suppress animal enteritis. *Bifidobacterium* and *Lactobacillus* species possess the capacity to magnify the impact of radiotherapy. These microorganisms functionally stimulate anti-tumor immune responses and mitigate tissue inflammation, potentially converging with the effects of radiation therapy for rectal cancer [47].

Given the similarities between cervical and colorectal cancer in terms of irradiation sites and modalities, Sims et al. unexpectedly discovered that patients with high intestinal flora diversity at baseline had a more significant decline in intestinal flora diversity from the start to the fifth week of CRT than patients with low intestinal flora abundance at baseline. This finding suggests that the optimal target group for chemoradiotherapy intervention may be patients with high baseline gut richness and diversity rather than those with low. It was shown that abundant microbial diversity had increased activation of CD4+ lymphocytes infiltrating cervical tumors and CD4 cell subpopulations expressing Ki67 and CD69+ during radiotherapy [53].

## 3. Discussion

**Figure 5:**
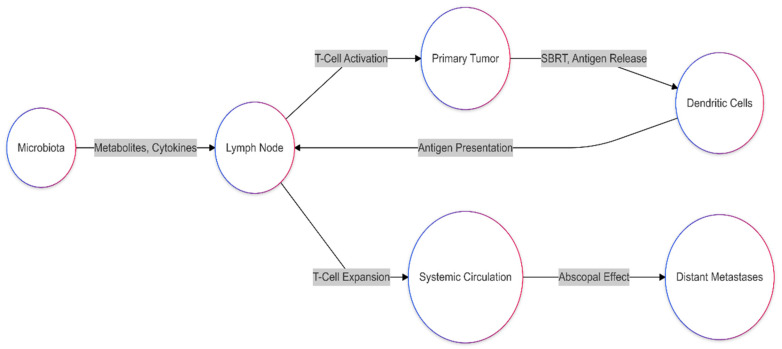
The figure depicts how changes in the gut microbiota influence an abscopal effect via SBRT for a systemic antitumor response. Microbial conditions activate the priming of immune responses in the lymph nodes by releasing cytokines and microbial metabolites. Meanwhile, the local deletion of tumor cells by SBRT releases tumor-associated antigens taken up by dendritic cells and cross-presented to CD8 cells. These activated T-cells undergo proliferation and expand into the systemic circulation, manifest cytotoxicity against the primary and non-irradiated distant tumor sites, and finally lead to the abscopal effect.

An important area of focus in oncology is the complex relationship between the gut microbiota and RT results. Growing data highlight the microbiota’s ability to affect epithelial healing, mediate inflammation, and modify host immunological responses—all of which are critical in determining the toxicity and effectiveness of RT (Table 2). Under RT-induced oxidative stress, beneficial microbial metabolites such as butyrate have shown protective effects in maintaining the integrity of the gut mucosa [6,14]. Cazzaniga et al. showed that supplementation with butyrate-producing probiotics significantly reduces gastrointestinal toxicity during RT and has been associated with immune homeostasis repair [54]. The dysbiosis condition, with reduced microbial diversity and promotion of pathogenic species, aggravates the RT toxicity of diarrhea and mucositis. For esophageal cancer, the reduced gut microbiome diversity has been linked to poorer neoadjuvant chemoradiotherapy responses. At the same time, a higher abundance of *Lactobacillaceae* corresponds to improved inflammatory and nutritional indices and better treatment outcomes [55,56,57]. Similarly, gut microbiota metabolites such as indole-3-acetic acid may influence pancreatic cancer outcomes, inhibiting tumor cell autophagy and reducing tumor stress adaptation during therapy [9,57,58]. In rectal cancer, specific microbial taxa, including *Clostridium sensu stricto 1* and *Intestinimonas,* have been linked to better nCRT responses, suggesting microbiota modulation’s predictive and therapeutic potential [59,60]. Findings also indicate that FMT may enhance dendritic cell activation and CD8+ T-cell infiltration into the tumor microenvironment, potentially augmenting the efficacy of radiotherapy. However, further clinical validation is required to determine optimal FMT protocols and patient selection criteria for its integration into standard oncological practice. For clinical application, FMT would require careful donor selection to ensure the safety and efficacy of microbiota transfer [18,27,49]. Ideal donors would be healthy individuals with a diverse and balanced gut microbiota, free from transmissible infections and chronic diseases. Standardizing donor screening, including stool microbiome profiling and pathogen exclusion, would be critical to minimizing risks. Additionally, the mode of FMT administration—whether via oral capsules, colonoscopic infusion, or enema—would need to be optimized based on patient-specific factors, such as the tumor location and prior treatment history [51]. Considering the complexities of direct microbiota transplantation, alternative approaches like the use of defined microbial consortia or postbiotic metabolites may provide a more controlled and reproducible strategy for microbiota modulation in RT settings [20]. Emerging evidence suggests that intratumoral bacteria may influence tumor response to RT by modulating DNA repair pathways, immune evasion mechanisms, and inflammatory signaling within the tumor microenvironment. However, these associations require further investigation to determine causality. For example, *Fusobacterium nucleatum* has been associated with increased radioresistance, potentially by upregulating DNA repair enzymes such as poly (ADP-ribose) polymerase (PARP) and activating autophagy-related signaling pathways, including AMP-activated protein kinase (AMPK) and mammalian target of rapamycin (mTOR), which promote tumor cell survival under RT-induced stress [42,46]. On the other hand, *Bifidobacterium* has been linked to enhanced dendritic cell activation and T-cell priming, which may improve systemic anti-tumor immunity and RT efficacy [61,62]. Moreover, it is becoming increasingly clear that the intratumoral microbiota can directly influence tumor response to radiotherapy by modulating immune responses, DNA repair processes, and various cellular signaling pathways [17,63]. This is particularly relevant for SBRT, which delivers high doses of radiation over a short timeframe. The working hypothesis is that in addition to mechanisms already mentioned—such as *F. nucleatum’s* capacity to enhance DNA repair or *Bifidobacterium’s* immunostimulatory effects—the ablative radiation doses used in SBRT may further intensify the local inflammatory response within the tumor. As a result, the role of the intratumoral microbiota in determining treatment outcomes becomes even more pronounced [2,42]. For example, high-dose radiation can trigger the release of tumor neoantigens and immunogenic “danger” signals (DAMPs), thereby recruiting and activating various immune cells in the tumor microenvironment. If a “favorable” population of intratumoral bacteria (such as certain *Bifidobacterium strains*) is present, it may further stimulate dendritic cells, enhance antigen presentation, and bolster T-cell responses. This immunological cascade can culminate in the “abscopal effect”, which strengthens systemic anti-tumor immunity and can target metastatic lesions [46] (Figure 5). In contrast, “unfavorable” bacterial species, such as *Fusobacterium nucleatum*, may bolster DNA repair mechanisms in tumor cells, diminishing their sensitivity to high-dose radiation and potentially compromising SBRT efficacy [48,64]. While existing evidence strongly suggests a modulatory role of the intratumoral microbiota in SBRT response, direct causal relationships remain to be fully elucidated. Questions still abound regarding how different bacterial strains interact among themselves and with key cellular signaling pathways and to what extent we can manipulate this microbiota—whether through the use of probiotics, antibiotics, or FMT—to improve SBRT outcomes. Further mechanistic studies in this area could pave the way for personalized treatment approaches in which the intratumoral microbiota is strategically modulated before or during SBRT to maximize therapeutic benefit.

### Limitations

This review has several limitations. First, the heterogeneity of the gut and intratumoral microbiota among patients poses a challenge in drawing generalized conclusions. Individual variations influenced by diet, genetics, and prior treatments may impact microbiota composition and its interaction with radiotherapy. While preclinical and early clinical studies suggest promising microbiota-targeted interventions, the lack of standardized protocols, optimal dosages, and long-term safety data limit their current clinical application. Additionally, the causal relationship between microbiota composition and radiotherapy outcomes remains incomplete, requiring further mechanistic and longitudinal studies. Finally, most available data are based on small cohort studies, underscoring the need for larger, well-designed clinical trials to validate the therapeutic potential of microbiota modulation in radiotherapy.

## 4. Conclusions

Modifying gut flora in GI tumors seems a promising method to augment the effect of RT treatment. Through an improved immunologic response, decreased inflammation, and reinforcing the gastrointestinal barrier, microbiota-targeted interventions have the potential to reduce RT-associated adverse events and enhance therapeutic outcomes. However, patient responses may vary depending on individual microbiota composition, tumor type, and immune status. The biological complexity of the microbiota further requires careful patient selection, personalized strategies, and rigorous clinical validation despite the variance in preclinical and early clinical findings.

## 5. Future Directions

Future studies should concentrate on personalized strategies for microbiota modulation based on individual patient profiles, comprising unique microbiome composition and functional dynamics. Large clinical trials to validate the efficacy and safety of probiotics, prebiotics, and other microbiota-targeted therapies would allow for establishing the optimal strain, dose, and duration of treatment. In particular, future research should determine the optimal donor selection, number of FMT procedures, and timing for microbiota transplantation in patients undergoing RT to mitigate toxicity and enhance therapeutic efficacy. Furthermore, the integration of artificial intelligence into microbiota-based precision oncology holds tremendous potential for enhancing personalized treatment strategies. Advanced machine learning algorithms can analyze complex microbiome datasets to identify predictive biomarkers that correlate with treatment response and toxicity, enabling clinicians to tailor radiotherapy regimens based on an individual’s unique microbial profile. Additionally, exploring the roles played by the microbiota in promoting new radiotherapy modalities like FLASH radiotherapy and proton beam therapy may hold great promise in protecting against toxicity and augmenting therapeutic response rates. Attention should also be given to the long-term persistence of the microbiota following therapy and possible long-term risks associated with microbiota modulation. Ultimately, integrating microbiota-targeted therapies into routine oncology practice will require a combination of personalized strategies, rigorous clinical trials, and regulatory standardization to maximize therapeutic effectiveness while minimizing risks.

## Figures and Tables

**Table 1:** Summary of key studies on the gut microbiota and its impact on chemoradiotherapy outcomes in esophageal cancer.

Study	Participants (n)	Cancer Type	Treatment	Diversity Impact	Key Bacteria
Van den Ende et al. (2024) [27]	172	Predominantly adenocarcinoma	Neoadjuvant chemoradiotherapy	Lower diversity = poorer survival	*Fusobacterium* (poor outcomes)
Sasaki et al. (2023) [28]	51	Squamous cell carcinoma	Chemoradiotherapy	Higher diversity = better outcomes	*Lactobacillaceae* (favorable response), *Fusobacteriaceae* (advanced stages)
Li et al. (2024) [30]	31	Squamous cell carcinoma	Chemoradiotherapy	Higher diversity = better outcomes	Not specified
Jiang et al. (2021) [31]	32	Not explicitly stated	Chemoradiotherapy	Higher diversity = better outcomes	*Bacteroides, Faecalibacterium* (favorable response)

**Table 2:** Key bacterial taxa influencing radiotherapy and chemoradiotherapy outcomes.

Bacterial Species	Mechanism of Action	Key Virulence (or Virulence-Associated) Genes	Impact on Radiotherapy/Chemoradiotherapy
*Bifidobacterium* spp.	Produces short-chain fatty acids (e.g., butyrate); enhances dendritic cell activation and T-cell priming	*Sortase*-dependent pili genes (in some strains) for mucosal adhesion Generally not considered pathogenic	Boosts anti-tumor immune responses; improves treatment efficacy; and reduces toxicity
*Faecalibacterium* spp.	Produces butyrate; promotes regulatory T-cell differentiation and exerts anti-inflammatory effects	No well-characterized virulence genes in common strains	Associated with better clinical outcomes and diminished RT-induced toxicity
*Lactobacillus* spp.	Supports gut barrier integrity; produces anti-inflammatory metabolites; and modulates immune responses	Some strains possess *spaCBA* pilus clusters for adhesion Typically non-pathogenic; few “classic” virulence factors	Mitigates RT-induced inflammation and mucosal injury; enhances chemoradiotherapy responses
*Fusobacterium* nucleatum	Activates pro-inflammatory pathways (NF-κB); promotes DNA repair and autophagy	*fadA* (adhesion/invasion) *fap2* (immune modulation, adhesion)	Contributes to radioresistance, potentially leading to poorer treatment responses
*Akkermansia muciniphila*	Stimulates Toll-like receptor signaling to boost IFN-γ production and antigen presentation	Outer membrane proteins such as *Amuc_1100* (important for mucosal interaction) Limited recognized “virulence” factors in humans	May enhance immune-mediated responses during RT/chemoradiotherapy
*Clostridium sensu stricto 1*	May produce beneficial metabolites that support immune homeostasis and modulate local inflammatory responses	Toxin genes vary by species (*cpa* alpha toxin in specific *C. perfringens* STs) Virulence plasmids in some members	Correlated with favorable responses to neoadjuvant chemoradiotherapy in rectal cancer
*Intestinimonas*	Produces short-chain fatty acids; supports mucosal and immune homeostasis	Not well characterized in terms of virulence genes Generally regarded as commensal/beneficial	Linked to improved response in patients undergoing neoadjuvant chemoradiotherapy
